# Training medical students in human rights: a fifteen-year experience in Geneva

**Published:** 2012-09-30

**Authors:** Philippe Chastonay, Véronique Zesiger, Jackeline Ferreira, Emmanuel Kabengele Mpinga

**Affiliations:** 1 Unit of Development and Research in Medical Education, University of Geneva Faculty of Medicine, Geneva; 2 Institute of Social and Preventive Medicine, University of Geneva Faculty of Medicine, Geneva; 3 Swiss School of Public Health Plus, Zürich

## Abstract

**Background:**

Training health professionals in the field of human rights has long been advocated by the United Nations. Over the past decade some medical schools have introduced health and human rights courses, yet by far not all. This paper describes the objectives and the content of the Health and Human Rights program developed at the Faculty of Medicine, University of Geneva.

**Methods:**

The health and human rights program was developed through the identification of the course objectives, contents, and educational modalities using consensus techniques, and through a step by step implementation procedure integrating multiple evaluation processes.

**Results:**

Defined objectives included the familiarization with the concepts, instruments and mechanisms of human rights, the links between health and human rights, and the role of health professionals in promoting human rights. The content ultimately adopted focused on the typology of human rights, their mechanisms of protection, their instruments, as well as social inequalities and vulnerable groups of the population. The implementation proceeded through a step by step approach. Evaluation showed high satisfaction of students, good achievement of learning objectives, and some academic and community impact.

**Conclusions:**

High interest of students for a human rights course is encouraging. Furthermore, the community projects initiated and implemented by students may contribute to the social responsibility of the academic institution.

## Background

Over the past decades many medical schools have implemented major curriculum reforms^1^–^3^ addressing organizational aspects (access to medical studies, selection procedures), educational approaches (problem-based learning, e-learning) or specific contents (health of minorities, new technologies, palliative care).^4^

At the Faculty of Medicine, University of Geneva, an important curriculum reform was introduced in 1995 with three major orientations: a problem-based learning approach in basic medical sciences, an early familiarization of students with basic clinical skills, and an early and strengthened exposure to community health issues.^5^

Among the community-oriented training activities a program focusing on health and human rights was implemented according to international recommendations.^6^,^7^

Although training health professionals in the field of human rights has been advocated by the United Nations,^8^,^9^ the situation in medical schools could still be improved as suggested by various authors.^10^,^11^ Yet several studies show that human rights training in medical schools is not very intensive nor well anchored. Claudot reports that in the European Union, 63% of the 70 participating faculties of medicine had some teaching on human rights, generally as part of an ethics course.^12^ Cotter mentions that 32% of 125 medical schools in the USA have some kind of human rights course.^11^ Numerous elements might explain these situations. To mention two of them: first, human rights have only recently been clearly identified as a major health determinant in the context of the HIV pandemic;^13^ second, the lack of a critical mass in terms of research and training in the field of discrimination, stigmatization, and violation of basic human rights.^14^

Furthermore, teaching human rights in a medical school can be rooted in basic human rights violations reported at the Nuremberg trial or in human rights issues raised by the HIV/AIDS pandemic (equal access to medication, stigmatization of patients and their relatives, discrimination of infected people). It can also refer to recommendations of professional associations. Yet new social developments such as easy access to information by patients and their families, aging of the population, and increasing financial burden in the health sector must also be considered.

Our article describes the objectives and the content of the Health and Human Rights program developed at the Faculty of Medicine, University of Geneva. It also presents factors that have contributed to the inclusion of the course into the curriculum, the implementation procedure with its difficulties and opportunities, as well as some evaluation data over a fifteen-year span.

## Methods

The identification of the course objectives, contents, and educational modalities was done using several approaches: 1) various methods of consensus among teachers and public health professionals (visualized discussion, nominal group, directed brainstorming); 2) a review of the contents of existing courses;^10^ and 3) a survey among medical students.^12^ A progressive implementation process was adopted in the context of a global curriculum reorganization, and facilitating or obstructing factors were monitored. The global evaluation process of the program included several elements. First the perception of students was monitored on a 5-point Likert scale (1=very dissatisfied; 5=very satisfied) and addressed items such as the quality of the organization, the clarity of the objectives, the usefulness of the course material, the preparedness/motivation of teachers, the quality of the hand-outs, as well as general satisfaction. Second, the achievement of learning objectives was measured through certifying examinations, such as a multiple-choice exam, a written report, or a presentation of group work to fellow students*.*

## Results

First, the conceptual basis of the link between health and human rights were identified (Table 1). This served as a base for the definition of the objectives, contents, and educational approach of the course. Consensus was obtained on several objectives, i.e., students should be able to describe (and eventually apply) the philosophy, instruments and mechanisms of human rights, the nature and strength of links between health and human rights, and the role of health professionals in preventing basic human rights violations. Table 2 lists the topics addressed in courses and seminars.

Second, on a consensus basis among training staff several educational approaches were adopted, such as classical didactic lectures, testimonies of patients, report of field experiences by professionals, seminars based on assigned group work to students, and community visits.

Third, implementation was progressive. During the initial period (i.e., as early as 1996) the Health and Human Rights course included a series of eight lectures included in a public health course for 3rd year students, focusing on social exclusion. In a second phase (starting in 2002) a one-month, full-time elective course was developed for 5th year students, focusing on discrimination the most vulnerable groups face in the health system. In a third phase, when the first year curriculum was reorganized (in 2005), a three-hour introductory course on Health and Human Rights was introduced. Finally, when the Faculty adopted the Bologna Guidelines for higher education (in 2007), an one semester elective course (3 ECTS) on Discriminations, Health and Globalization was developed for 2^nd^ and 3rd year students. Furthermore, master thesis subjects were proposed for 5th year medical students.

Fourth, in our Geneva context, there have been several facilitating elements that allowed us to develop a Health and Human Rights program, i.e., the implementation of a major curriculum reform adopting problem-based learning and thoroughly redefining the learning objectives; a receptive local hierarchy due to the long humanitarian tradition in Geneva; the support of the Geneva Academic Society in funding the University Forum “Health and Human Rights”; the support of the Geneva International Academic Network allowing the development of research in the domain “health and human rights”; the encouragement of Geneva-based international organizations active in the field of human rights and humanitarian rights, which allowed the development of close collaborations. Hurdles were few: some scepticism among basic science teachers and limited long-term resources allocated to the program.

Finally, the evaluation focused on student’s perceptions and achievement of learning objectives. Students’ perception of the Health and Human Rights teaching (agglomerated data from 1996 to 2011, including 896 completed questionnaires with an overall response rate of 68% of 3rd to 5th year students) shows a high level of satisfaction over the fifteen-year span. On a 5-point Likert scale (1 = very dissatisfied, 5 = very satisfied) the mean of the global appraisal of the course is 4.4; the organization gets 4.4, the clarity of objectives 4.4, the preparedness/motivation of teachers 4.8, the usefulness of hand-outs 4.3. Based on various examinations, the achievement of learning objectives has been estimated by the coordinating teaching staff to be excellent for 42 % of students over the years, good for 44%, and fair for 14%.

## Discussion

Recommendations in relation to human rights training targeting medical students^15^ propose that, since “every human being is born free and equal in dignity and rights” each medical doctor recognizes “the separate, inviolate nature of the individual person who will face him/her in the casualty area, the examination room, the office, the conference room”. This guided us in implementing a Health and Human Rights program at our institution. First, content was defined on a consensus basis among teaching staff and seemed to fit in with the clinical and social environment and coherent with other educational initiatives.^16^ Second, multiple educational strategies were adopted as recommended in the literature,^17^ which probably contributed to the positive perception students had of the program. Third, the progressive implementation allowed to overcome potential ‘logistic bottlenecks’ and ‘obstacles to change’ as well as the support of ‘faculty allies’, as some authors have reported.^18^ Fourth, the global human rights context in Geneva was a facilitating factor for implementing the program without too many difficulties, even though human rights might not be the top priority of the medical school. Finally, the high level of satisfaction in our study is not a surprise as mentioned in the literature,^19^ since several of our courses are electives, thus corresponding to a student’s choice. The evaluation of the program by students, who globally performed very well on the certifying evaluations, stayed positive over the years, perhaps reflecting their enthusiasm,^20^ more than the quality of the program itself.

## Conclusion

The fifteen-year experience at the Faculty of Medicine, University of Geneva, with a Health and Human Rights course has shown the interest of students for such a course as well as their commitment to human rights. Furthermore, the community initiatives that students have implemented may have contributed to the social responsibility of the academic institution. Finally, it seems to us, as Ewert et al. have also noted,^18^ that it is imperative that information and experiences be shared with others in the health and human rights educational community.

## Figures and Tables

**Table 1:** Links between health and human rights: conceptual basis

- ***Health is a question of human rights****,* which implies obligations to States to see to an appropriate organization and functioning of the health systems, to the availability and accessibility of health services, to the quality of health care and to its acceptability (the General Comment 14/2000 on the right to health of the UN-Committee on Social, Economic and Cultural Rights). - ***Violation of basic human rights affect the health of individuals and communities****,* since they may constitute hurdles to the access to health care and health services; when violations of basic human rights are extreme, such as in torture, they might generate complex physical and mental health problems even over several generations and cause high social and economic costs. - ***A better protection of human rights contributes to the protection and promotion of health****,* e.g. respecting the right of association and the liberty of peaceful assembly facilitates the emergence of associations, foundations and other non-governmental organizations which potentially play a role in defining and implementing health policies; e.g. gender discrimination in access to education potentially deprives the health sector of qualified workforce, thus decreasing the quality of health care, its availability and its accessibility. - ***Public health tools are necessary for the protection and promotion of human rights****;* the protection of human rights requires a system that allows the monitoring of their implementation: collecting reliable data and analyzing it with scientific rigor allows to evaluate the effectiveness and the justice of human rights; furthermore, health prevention and promotion strategies can stimulate and enrich human rights protection and promotion programmes. *Adapted from: Nygren, K.H. 25 questions and answers on Health and Human Rights, WHO, Geneva, 2002, 36p.*

**Table 2:** Health and human rights course and seminar contents

Course contents	Seminar contents
Social inequalities and health Typology of Human Rights Mechanisms of protection of Human Rights National and international Human rights instruments Violence against women Access to health care of migrants and refugees Discrimination of elderly in the health system Persons living with HIV/AIDS Stigma and discrimination in the field of mental health Children living with a disability	Poverty and Health Social exclusion and Health Social insurance coverage and non-discriminatory access to health care Illiteracy and access to health Health in prisons Access to health care of clandestine workers Health and Prostitution Torture: impact on health of victims and role of health professionals Genital mutilation and universality of basic human rights Victims of landmines and related care

## Appendix 2 Example of a certifying exam

(case study to be completed in a half-day, open-book exam)

**EXAMEN DU COURS A OPTION**

***«Discriminations, Santé et Droits de l’Homme»***

### Etude de cas Fictive

***Mise en place d’un Observatoire cantonal de lutte contre les discriminations***

#### a) Contexte

Préoccupé par les discours et pratiques discriminatoires dans le canton de Vaubourg, le conseil d’Etat a chargé l’Université de Genève de lui proposer un outil de monitoring des discriminations dans le canton.

L’Université de Genève vous recrute comme expert(e) et vous charge de l’exécution de ce mandat.

#### b) Travail demandé

Partant de votre expérience, des données politiques et sociales:

- Analyser les problèmes sous l’angle épidémiologique (déterminants sociaux, culturels, économiques, facteurs de risques et conséquences);
- Identifier les droits fondamentaux susceptibles d’être atteints, et les conventions internationales qui les protègent;
- Identifier les droits fondamentaux qui sont bafoués;
- Esquisser un projet d’observatoire des discriminations en Suisse (Sa mission, ses objectifs, son organisation);
- Indiquer, sur la base des obligations de la Suisse en matière de protection et promotion des droits humains, en particulier les instruments internationaux ad hoc, une liste non limitative des INDICATEURS de Suivi des discriminations;
- Présenter votre travail devant les membres du Rectorat de l’Université de Genève et les Responsables du Service de Lutte contre les discriminations du Département cantonal de Justice et Police.
